# Abstracts and Gleanings

**Published:** 1877-08-20

**Authors:** 


### Abstracts and Gleanings.

### CRANIOTOMY.

The following cases of craniotomy were reported to California State Medical Society, Dr. Cushing, of Oakland:

*Case* 1—Jane T., aged 37; native of Ireland; married three years; was taken in labor with her first child, May 31st, 1871, at noon.

Dr. J. C. Van Wyck was her medical attendant, and upon examination found the os somewhat rigid, and the brim of the pelvis somewhat contracted antero-posteriorly.

The labor progressing slowly, the following morning I was called to see the case in consultation.

I found the os uteri fully dilated, and upon introducing my hand into the vagina, and up to the brim of the pelvis, the condition was found as follows: the promontory of the sacrum projected so far into the pelvis inlet as to leave the antero-posterior measurement, according to my estimate only two and a half inches; the head of the child was somewhat impacted and was flattened out to a considerable degree by the powerful action of the uterus in the efforts at expulsion.

Upon consultation it was deemed best to perform craniotomy and Dr. Van Wyck, by means of Thomas' perforator, the crochet, and the craniotomy forceps, succeeded after an hour's hard work in delivering the woman.

The patient was rendered insensible to pain by the use of chloroform and the effect afterwards kept up by ether.

She rallied quite well from the operation, and ultimately made a perfect recovery, although she suffered much from a large pelvic abscess which kept her in bed for over two months.

*Case* 2—Honorah D., aged 30; native of Ireland; married 5 years; has given birth to three children, one born at eight months, living, two born at full term, dead.

I was called on the e[illegible]ing of April 25, 1873, to see the above patient, and found her suffering severe labor pains. She was in charge of a German midwife, who assured her that if she would bear down, she would soon be through with her labor. The woman had been in labor about twelve hours, and according to her calculations was at her full term.

Upon examination, I found the head presenting, and a good deal flattened, the promontory of the sacrum projecting in a marked degreed, and rendering the passage of the head impossible without lessening its size. Dr. Bradway saw the case with me, and we decided upon craniotomy as the only resource.

After evacuating the bladder and bowels, I placed the woman upon her back with the hips near the edge of the bed, and proceeded to perforate the head with Thomas' perforator. I then broke up the brain substance freely, with the perforator, and removed a considerable portion with my fingers. I then applied the large craniotomy forceps, one blade being introduced within the opening in the head, and the other outside the scalp, and by slowly but firmly twisting the bones, succeeded, in the course of a few moments in so breaking up and dislocating the bones which form the arch of the cranium as to leave the upper portion of the head in a jelly like mass.

I then, seizing the occipital bone, made traction and had no difficulty in effecting a delivery.

The operation lasted half an hour. As chloroform was seriously objected to, none was given, the patient remaining submissive and quiet. I estimated the antero-posterior measurement at 2½ inches. The patient was small and slight built.

She made a perfect and rapid recovery, and called upon me at my office in less than three weeks after delivery.

*Case* 3—Mary Ann G., aged 36; native of Ireland; married 6 years; has given birth to two children, both still-born, and delivered with forceps.

Was called to attend her on the night of May 7, 1875, at 9 pm.

She had been attended during the afternoon by two physicians who had made repeated efforts to deliver her with forceps, but had failed, the patient stating to me that the instruments constantly slipped off, causing her great pain. The attending physician suggested craniotomy, but the Catholic priest would not consent to the procedure, as being con trary to the rules of their church ; thereupon the medical attendants left the house.

Upon examination, I found the soft parts much swollen and inflamed by the repeated use of forceps ; the head presented normally, but the conjugate diameter was much lessened, as in the last case, the antero-posterior diameter uot exceeding 2¼ inches.

After consultation with Dr. J. C. Van Wyck, it was decided that but one resource held out hope, and that was lessening the size of the child's head, one thing being certain, whatever was done must be done at once, as the woman's pulse was rapid and feeble. She already had lost a large quantity of blood, and had an extremely anxious expression on her face.

Assisted by Dr. Van Wyck, chloroform was administered, and craniotomy performed in the same manner as the last case, but attended with more difficulty, owing to a greater amount of deformity ; even after the head was delivered, it required the use of a great deal of force to get the shoulders through the upper strait. The operation occupied one hour. The patient rallied well under the influence of morphia and whisky, and has made a slow but sure recovery.

In reporting the cases of craniotomy, a few points are to be noticed :

First, that in the last two cases, where the women had previously given birth to children with the aid of forceps, each succeeding labor was attended with more and more difficulty, showing that there was an advancing pelvic contraction.

Second, that after the head has been perforated by the Thomas perforator, that no more efficient instrument could be desired than a pair of heavy-jawed craniotomy forceps, for with these the whole cranial vault can be broken up and rendered as compressible as a bag of jelly, by introducing one blade into the cavityof the skull and applying the other upon the outside of the scalp, the whole cranium can be broken up, by gradually changing the position of the forceps from one portion of the head to another

The soft parts of the mother are usually protected from the fragments of bone, by the scalp, which is left intact, except at the point of perforation.

I have found the most advantageous point to seize for the purpose of making extraction, is the occipital bone, for by this means the chin is thrown down upon the chest and the least possible resistance is met with in extraction.

### A SIGN OF EARLY PREGNANCY.

Dr. Eugene C. Gehrung, in *Practitioner*, remarks :

The diagnosis of early pregnancy is beset with such great difficulties that any addition to its signs should find a ready welcome. Part of the value of the sign I shall describe is lost, however, in so far that it is especially useful to the gynecologist only. The latter is often imposed upon by unscrupulous women, who, on account of their unwillingness to raise children, or to undergo the unpleasantness of a pregnancy, and under pretext of some disease of the womb, would have him produce abortion unknowingly ; for which, if successful, he would certainly not receive her thanks, but could be sure to earn all the blame possible for his carelessness, and ridicule for his ignorance. It is easier to criticise than to avoid such mishaps. The history of these cases is often made up in a very deceptive manner.

Besides this not uncommon class fo cases, there is another consisting of women who, suffering actually from disease of the generative organs, are ignorant of their pregnant state; and who, by giving a history of their ailments that would leave the possibility, at least the probability, of a pregnancy out of the question, may place their attendant in a very unpleasant position by the results of his treatment, which, though unavoidable, with the present means of diagnosis at his disposal, would still subject him to the severest censure from the patient, the public, and the profession.

In the absence of any distinct signs of pregnancy, particularly when the history of the case appears good and other symptoms correspond with it, the practoner is very apt to use the uterine sound or probe for further information, diagnosis or treatment. It is at this stage where the benefit of the sign presently to be described becomes apparent.

Several years ago, when my attention was first attracted by this sign, and its meaning not being fully understood, I disregarded it to my great sorrow and mortification. Since then I have met with a limited number of cases in which I escaped a similar annoyance by honoring this sign with the regard due it, and by endeavoring to err rather on the safe side. These latter cases either went to full term, or were terminated to my knowledge by somebody else, who earned the merited or unmerited reproach, as the case may be.

If a sound or probe be introduced into a healthy womb in the direction of its axis, previously defined by the usual methods, the sensation communicated to the hand through the sound when touching the fundus, is that of touching a moderately solid object, much resmb ling that produced in touching the roof of the mouth with the same instrument. If an ovum of any size be present, circumstarces are changed.

The scund will proceed with equal ease to and through the internal os, but as soor [illegible] he ovum is touched the sensation communicated to the hand is like that felt in pushing the sound against a bladder filled with fluid, that is a gradually increasing resistance; in addition to which, according to the size of the ovum, or the amount of pressure exercised, the sound will be driven back when loosely held with a greater or less amount of force communicated to it by the tendency of the ovum to resume its former globular shape. In other words, the sound meets with an exceedingly elastic body beyond the os internum instead of the solid uterine walls. If, at the same time, the sound enters beyond the normal distance, the probability of pregnancy—a further advanced pregnancy—is still greater. It is evident that whenever there is the slightest suspicion of pregnancy, the introduction of the sound should be made very gently, and as this entails no appreciable loss of time or extra trouble, this precaution may be used in every first examination with that instrument; or rather, should always be used, as patients may, and frequently do, get pregnant while under treatment.

This system, when present alone, is not positive evidence that pregnancy exists, nor when absent, negative; because there are a number of other conditions which give a similar result. On the other hand, it is a well known fact that the ovum, in the earlier periods of pegnancy, is only attached to a greater or less part of the interior of the womb, and consequently the sound may slip by it without producing that particular effect; yet when found it should caution the operator, and make him reconsider the case carefully before proceeding further.

Differentiation is necessary only between products of conception on the one hand, and certain pathological conditions on the other. These latter are:

Uterine flexions, Retained placenta, Uterine polypi, Retained blood clot, and Carcinoma of the body of the womb.

### ENTERIC FEVER—IS IT CONTAGIOUS?

We extract from the Paper of J. S. Knox, M. D., to the American Medical Association, the following remarks:

In order to study the etiology of enteric fever, the writer addressed a series of questions to physicians of eminence in different parts of the Union, especially the State of Virginia. Many of the replies were contradictory. After defining the difference between direct or miasmatic contagion, positive cases and opinions were quoted from these replies, for and against direct contagion. The conclusion reached was, that while the evidence of direct contagion was often incomplete, there was positive proof of contagion from miasmatic influences: viz., infected water in the air. Many positive opinions were also quoted of the *de novo* origin of the disease, and the cases cited. Still, against such an origin it may be said that the disease may occur under favorable conditions, where direct importation may be excluded; thus, from germs left long ago, or from germs latent in the body and developed by favorable circumstances, or by wide diffusion in the air, of desicated but living germs.

As a summary from the answers received, the following may be stated as external conditions favorable to the development of enteric fever:

1. Excremental filth undergoing moist decomposition, and contaminating air and water.
2. Contaminated milk.
3. Vegetable decomposition.
4. Decay of dry timber.
5. Soil saturated with organic impurities independently of water contamintion.
6. Contaminated water.
7. Some undefined telluric influence not associated with organic contamination.

In regard to the relations between typhoid and malarial fevers, a majority of the correspondents consider them distinct, and of separate origin. A few recognize hybrids. Most consider typo-malarial fever to be an adynamic form of malarial fever, due to pythogenic influences. Some recognize a decided antagonism between the two, and quote the marked absence of typhoid in malarious regions, and if the former does occur it is of mild type. The drying up of a marsh producing malaria, will introduce a new type of fever, viz: typhoid. Abundant illustration of this is seen in Illinois and the South. May be due to the fact that malaria requires a saturated soil, and typhoid a desicated one.

English theories exclude epidemic influences, yet apparently typhoid fever may extend in the tracts of atmospheric currents independent of direct intercourse. Indeed, the observation of some give it a migratory character, due neither to local causes, nor yet to direct contagion. Further observation is necessary to establish many of the above conclusions. To do this, isolated cases must be studied, and meteorological conditions during the presence of an epidemic must be observed.

In the discussion that followed, Dr. Comyges, of Cincinnati, reported a succession of cases in his own family, proving the infectious character of enteric fever.

Dr. Plummer, of Rock Island, insisted that typhoid and malarial fever can occur at the same time in the same patient; proven by post-mortem examinations. There were many such specimens in the Washington Museum.

### JABORANDI.

Dr. Hutchins, in the Proceedings of the Medical Society of the County of Kings, says: To ascertain whether jaborandi was antagonistic to belladonna in respect to its influence on the secretion of milk, Drs. Ringer and Gould instituted experiments in two cases. "We administered thirty grains of jaborandi to a woman thirty-eight years old, confined of her ninth child four months previously. During suck–

ling she had very little milk, and the quantity had become much less of late. We gave her the medicine at ten A. M. She had suckled her child seven hours before. In ten minutes the drug produced its usual symptoms; in half an hour her breasts, which previously were flaccid, became tumid and distended, and on pressure yielded considerably more milk. In forty minutes the increase was still more marked, jetting forth in four or five streams.

To another woman, aged twenty-five, whose child is thirteen months old, we gave two doses of thirty grains, as the first had no effect. She emptied her left breast every ten minutes by pressure, and each of the three first emptyings yielded 40 minims. As soon as the perspiration and salivation became free, the quantity rose to 80 minims. The next time yielded 100 minims, the following 155 minims, the next time 80 minims. The salivation and perspiration at this time ceased. The next observation yielded 125 minims, the next 87 minims, the next 70 minims and the last 40 minims."—*Lancet*, January 30, 1875.

Dr. Will details the case of a mother who had been unable to suckle her previous child on account of the want of milk, and whose breast, on the fourth day after delivery, despite all efforts, were perfectly flaccid, and on pressure not a drop of milk could be obtained. Half ounce doses of the decoction were given, 2½ drams to six ounces water, three times a day, a strong decoction being at the same time applied to the mammæ. After two doses milk appeared. It continued to increase in quantity, and in ten days the drug was discontinued, as the secretion seemed to be fairly established. The child has every appearance of being well-nourished.—*British Medical Journal*, September 15th, 1876.

Dr. Bartholow used a fluid extract successfully in a case of deficiency in the secretion of milk in a nursing woman. "As the milk glands correspond in structure to the sudoriferous glands, and are merely differentiated and specialized for their particular office, the effects of this drug in increasing the production of milk might have been, *a priori*, expected."—*Materia Medica and Therapeutics*, p. 387.

JABORANDI AND BELLADONNA.

Though jaborandi and belladonna are in so many ways antagonistic, it has not been found that pilocarpine proves of any benefit in belladonna poisoning.

"The relation between belladonna and jaborandi is partly of analogy, but mainly of opposition. Jaborandi resembles atropia in quickening the pulse, flushing the face, and in exciting a more decided influence in adults than in children. On the other hand, it is diametrically opposed to atropia in its action on the salivary, sudoriferous, and mammary secretions, on the pupils, and on the minute arteries. Further, the tendency of belladonna to cause delirium contrasts with that of jaborandi to cause prostration and sleepiness."

The foregoing constitute nearly, if not quite all, the specific cases in which jaborandi has been employed, which have been reported in the journals up to this writing. There is good reason for this apparently meagre showing of interest.

Jaborandi is emphatically unique and distinct in its physiological effects, and, as was suggested in the opening sentence of this paper, it is to be estimated for its ability to fulfil certain conditions arising in a multitude of morbid states.

In this view it has been diligently studied, as witness the careful observations of Robin and Gubler, and the French pharmacologists; the elaborate examination of its properties by Ringer, Gould, Craig, and others in England; Ambrosoli's series of experiments with fifty hospital and private patients; Riegal, who wrote enthusiastically in its favor in an extensive treatise; Merkel, with his 20 cases from the Nuremberg Hospital; Lohrisch, with a large number of observations from the Clinical School of Frerichs; Penzoldt, with seventeen cases from the Clinical School of Erlangen; Rosenbach, with twenty-three cases from the School of Jena; Stumpf, with fifty-four cases from the Clinical School at Munich; Vulpian, Kohler and Soyka's experiments on animals, and the lately developed and growing interest among American observers. All these observers are in entire agreement as to the energetic dlaphoretic action of Jaborandi.—(*Deutschis Archiv. fur Klin. Med.*, November, 1875).

My own experience is entirely in harmony with the recognized properties of this drug, and I have had no failures to produce the prompt and active physiological actions with the jaborandi leaves obtained from Heydenreich Brothers, though these gentlemen say that some they had a year ago proved utterly inert.

The sum of the recorded experience is, that when diaphoresis is desired in any form of malady, it can be attained promptly and surely by jaborandi, while the size of the dose and the frequency of administration will regulate the profuseness and continuance of the diaphoresis, to meet any contingency desired.—*Proceedings Brooklyn Medical Society.*

### BROMIDE OF ETHYL AS AN ANÆSTHETIC.

At a recent meeting of the Academy of Sciences, M. Rabuteau gave some details of an investigation of the physiological properties and mode of elimination of bromide of ethyl.

Bromide of ethyl ($C_2H_5Br$), or "hydrobromic ether, is a colorless liquid, with an agreeable odor; it boils at about 40° C., has a density of 1.43, and burns with difficulty. The boiling point and density are, therefore, intermediate between those of chloroform and sulphuric ether.

Bromide of ethyl absorbed by the respiratory passages produces absolute anæsthesia as rapidly, or even more rapidly, than chloroform. This result has been established with frogs, rabbits, dogs, etc. After five minutes', sometimes after two minutes', inhalation, by means of a sponge saturated in bromide of ethyl, dogs are completely anæsthetized. The animals recover more rapidly than when chloroform is used.

When a solution of hydrochlorate of narceia, or hydrochlorate of morphia, was injected under the skin of dogs, before inducing anæsthesia, an action was observed analogous but perhaps inferior to the simultaneous action of narceia, or morphia, and chloroform.

Bromide of ethyl is not caustic, nor even irritant, compared with chloroform. It can be ingested without difficulty, and applied without danger, not only subcutaneously, but to the external auditory meatus, and to the mucous membrane. In this respect it is preferable to chloroform, which is very caustic, and to sulphuric ether, of which the ingestion is nearly impossible. Introduced into the human stomach in doses of one to two grammes, bromide of ethyl does not produce anæsthesia as when absorbed in sufficient quantity by the respiratory passages. It soothes pain, and does not disturb the appetite.

This anæsthetic is nearly insoluble in water. Nevertheless, water shaken with it acquires a pleasant taste and odor. Frogs placed in water so saturated undergo anæsthesia in ten or fifteen minutes.—*Druggists' Circular and Chemical Gazette.*

### SANTONIN.

In a short article on the use of this drug (*Med. Times and Gaz.*, July 7, 1877), Mr. E. Marlett Boddy says there is no doubt that santonin is, for many reasons, by far the most efficient anthelmintic which can possibly be administered to children, and its combination with calomel he has found to be most advantageous in every respect. Santonin, like every other therapeutic agent, requires care in its administration; and if it is allowed to remain in the system it acts deleteriously, like certain cumulative medicines. This pernicious after-action one of course seeks as much as possible to obviate, and the only way to do so, as regards santonin, is to combine it with some purgative, such as calomel, which carries it off.

According to Falck of Marburg, if santonin is allowed to remain in the system we get a substance called xanthopsin, into which santonin is supposed to be transformed under certain circumstances, which at present are not well ascertained. This xanthopsin is excreted by the urine, giving it a remarkable yellow color, causing a similtude to that secretion passed in jaundice, and its presence there is easily detected by caustic alkalies, which redden the urine. No doubt it is this xanthopsin which gives rise to those dangerous symptoms that have been so largely dilated on of late, and which many attribute to santonin only, forgetting or ignoring the presence of xanthopsin; and this mischievous action Mr. Boddy has found from experience to be entirely counteracted, or rather prevented, by administering calomel at the same time.—*Ab. Med. Science.*

---

TREPANNNIG FOR FRACTURE OF THE SKULL.—As a contribution to the literature of the operation of trepanning, Prof. von Linhart, of Wurzburg, reports the case of a young soldier, who was admitted into the Julius Hospital in 1866, suffering from a lacerated wound of the scalp and fracture of the parietal bone and of the squamous portion of the temporal bone. As the bone was not depressed and the symptoms were slight, the wound was merely dressed and the patient put to bed. On the second day twitchings in the muscles of the face and limbs were observed, which gradually became more severe, and on the third day well-marked epileptic attacks set in. These attacks lasted 10 or 15 minutes, and recurred almost every ten minutes. On the next morning the Professor trephined the patient as an exploratory measure, and found a small, pointed splinter from the vitreous table firmly imbedded in the dura-mater. This was seized with forceps and removed with some little difficulty. As there was but little depression of the fractured bone, no attempt was made to elevate it. The patient recovered quickly from the coma, and had no more epileptic attacks. Fourteen days after the operation he was able to be moved to his home.

In spite of this and some other successful cases, Prof. von Linhart does not believe trepanning, either primary or secondary, to be of such great value as is claimed by many writers. His opinion is based on the facts that he has seen many desperate cases recover without the operation, and on the other hand has been convinced by many operations and post mortems that trepanning alone will neither prevent nor cure a traumatic meningitis or encephalitis.—*Centralblatt fur Chirurgie*, May 19th.

TREATMENT OF CROUP BY EUCALYPTUS.—Dr. Walcker (*Gazette Medicale de Strasbourg*, January 1, 1877) treats pseudo-membranous laryngitis by tincture of eucalyptus globulus. He begins by an emetic of ipecacuanha, of which the dose varies according to age. This emetic is given morning and night once. He no longer employs tartar emetic in these cases, because it produces too much depression, and causes diarrhœa more often than ipecacuanha. This emetic relieves at the outset the gastric disturbance which ordinarily accompanies croup, calms the fever a little, and gives immediate relief. It can only act in this way, and it is incapable of expelling the false membranes. Two hours after the emetic he gives every hour a teaspoonful of a syrup composed of thirty-eight parts of simple syrup and ten parts of tinctureof eucalyptus for infants. He has given as many as fifteen to twenty teaspoonfuls in the case of a child six years old. When the patient sleeps at night, he should not be awakened. At the same time Dr. Walcker gives, as food, milk, coffee, eggs, and sopped brea . This alimentation is necessary; for cases of general diphtheritis or localized croup occur much more often in delicate children, with more or less scrofulous and lymphatic temperament and a feeble and delicate constitution, than in full blooded, strong, and robust children.—*Brit. Med. Journal.*

Chloral in Convulsions.—Dr. Jno. M. Thompson of South Carolina, says, in *Virginia Medical Monthly:* In a case recently of puerperal eclampsia, after blood-letting freely, I used large doses of chloral at short intervals with the most happy effects. In this affection it ranks with chloroform in controlling the convulsions, though it should be used freely in full doses of thirty to forty grains. In the spasm of children it is the remedy *par excellence*, and in numbers of cases in which I have used it, have never known it to fail.

Danger from the use of chloral hydrate has been apprehended by some, from it having been alleged to be converted into chloroform in the blood, but this idea has been pretty well exploded. I have never met with any deleterious effects after a constant use of the remedy for more than three years, and consider it equally as safe as any other agent of its class.

The profession should give it an impartial trial in this justly dreaded affection, and, after a trial, I am convinced they will always resort to it.

Properties of the Human Gastric Juice.—M. Charles Richet has been studying these matters upon the person of the patient on whom Verneuil successfully performed gastronomy. He has reached the following conclusions: 1. The acidity of the gastric juice, whether pure or mixed with food, is equivalant to 1.7 grammes of hydrochloric acid to a thousand grammes of fluid. 2. Acidity increases slightly at the end of digestion, and is independent of the quantity of liquid contained in the stomach. Wine and alcohol increase, but cane-suga diminishes it. 3. If acid or alkaline m tters are introduced, the gastric juice tends to return to its normal acidity. 4 The mean duration of digestion is from three to four and a half hours and more. Food does not pass successively but in masses. 5. According to four analyses made by a modification of Schmidt's method, it was proved that free hydrochloric acid exists in the gastric juice. 6. It is possible to extract all the lactic acid to nine parts hydrochloric acid. 7. Following the method of Berthelot, that is, by agitation with anhydrous ether and deprived of alcohol, it can be shown that lactic acid is free in the gastric juice. 8. The question so long in controversy as to the nature of the free acid in the stomach seems almost solved, and it may be said that in every 1,000 grammes of gastric juice there are 1.53 grammes of hydrochloric acid and 0.43 of lactic acid.—*Lyon Medicale*, May 13, 1877.—*Med. Record, No.* 3.

Profuse Sweating.—Dr. Finney used dilute sulphuric acid and liquor ferri perchloridi in phthisical sweating; also three grain doses of Dover's powder and sulphate of atropia (one-eighth of a grain for a dose) He mixed half a grain of the sulphate with sugar of milk, and divided the mass into forty pills. He bore witness to the value of atropia in the treatment of local sweating, and referred to a case of bromo-hyperidrosis of the feet, reported by Dr. Grimshaw (*Irish Hospital Gazette*, 1872, p. 52) as being cured by atropia. It had not, however, in his (Dr. Finney's) experience, checked the sweating in enteric fever.

Dr. MacSwiney had been taught to use the diluted mineral acids with bitter infusions, acetate of lead, and belladonna, as anhidrotics. Sponging the body with warm diluted vinegar was very efficacious. Having regard to the correlation of diarrhœa and perspiration in phthisis, he did not think it desirable to use energetic and continuous means of arresting diaphoresis in the third stage of the disease.

The chairman said that remedies like cod liver oil, which improved the condition of the system, often checked perspiration in a remarkable manner. Evening drinks should be forbidden, as far as possible. Cotton worn next to the skin and tepid sponging were useful. A powerful nervine like strychnia would lessen perspiration; but such remedies lost their effect after a time.—*Med. and Surg. Rep.*

COCOA AS A FOOD FOR INFANTS.—The great advantages to be derived from the employment of cocoa in the feeding of infants, especially of the poor, are obvious, for beside its heat-producing, flesh-forming ingredients, it is cheap, simple, and readily available. A teaspoonful, more or less, of a sound preparation of cocoa to half a pint of fluid, partly water and partly milk, even skimmed milk, when boiled for a minute or two, affords a wholesome meal to a hungry infant, and will *cœteris paribus* be thoroughly digested.

To present nutriment to the infant stomach, especially before the teeth are developed, in a perfectly fluid form, I have long since regarded as indispensable to the health of a child, inasmuch as the pepsin or solvent principle does not, as in adults, seem capable of reducing solids, not even pap, to such a state of solution that the lacteals or absorbent veins can act upon it with the same energy as in after life. The consequence is that the child, though largely fed is still hungry, accumulations take place in the intestines, its limbs and body waste as much from vitiated secretions, and the countenance assumes the canine ravenous expression of starvation and bad treatment.

I beg, therefore, respectfully to commend cocoa, as an article of infant's food, to the notice of my professional brethren.—*Wm. Faussett, Dublin Medical Press.*

STRICTURES.—In a valuable paper read before the Central, Ky., Medical Association, by Dr. L. S. McMurty, of Danville, we extract the following, which the author quotes from high authority:

(1) "All uncomplicated strictures, not highly irritable or resilient, should be treated by dilatation with soft instruments up to No 9, conical steel sounds afterward; reintroduction being made every fourth to eighth day—the older the stricture the longer the interval, as a rule, and intervals of one week being most serviceable in the majority of cases."

(2) "All strictures at or near the meatus must be cut."

(3) "Resilient, very irritable, and, as a rule, traumatic strictures are best treated by divulsion, if they lie below four and one-half inches from the meatus, otherwise by internal urethrotomy. When a resilient stricture cannot be divulsed, it should be cut—internally."

(4) "Impassable strictures may usually be overcome, where there is no restriction, by time, patience, and skill, with whalebone bougies. If, finally, found impassable, the treatment is external perineal urethrotomy."

A NEW METHOD OF TREATING NASAL CATARRH.—Dr. Arthur Hartmann, of Berlin, reports a new method of treating acute and chronic nasal catarrh, which he has found of great service. This treatment is of importance to the aurist, because middle-ear troubles are not infrequently caused by nasal catarrh. The author discovered that inflation with air, during the act of swallowing, not only mitigated the ear troubles, the deafness and roaring sounds, but also relieved the frontal distension and fullness of the head. The air is simply forced into the nose with a rubber balloon, after Politzer's method for the ear. In order to ascertain the effect of compressed air, in expressing fluids from the nose, the author made a number of experiments on dead bodies. He filled the cavities about the nose with fluids, and observed that when air was forced in the fluids were forced out. The author hereupon reports a number of cases in which the unpleasant symptoms of nasal catarrh were completely relieved in this way. To remove the crusts of ozena, he uses a brush fastened at right angles to the end of a thin flexible wire, an apparatus such as is used for cleansing tobacco pipes. The tenacious secretion is entangled upon the brush and removed. The nose is then washed out with water, and air is forced in after the manner described.—*Deutch. Med. Wochenschr., Cincinnati Lancet.*

ECBOLIC ACTION OF QUINIA.—Dr. Chiarleone presents, in the *Gaz. Med. Ital. Lombard.*, some few facts in relation to the alleged ecbolic action of quinia which have resulted from experiments and observations made by him in the Maternite of Milan. He dissents from the views of Dr. Chirone on the subject, published in *Lo Sperimentale* during 1874-'5. He declares that:

1. The sulphate of quinia is incapable of abbreviating the pause between one contraction and another, and hence can not augment the number of the latter.

2. The duration of the contractions before and during the action of quinia does not vary sensibly; the use of this drug would therefore be a disadvantage (perhaps from the sedative action exerted on the nervous system in general.)

3. Quinia, given near the time of parturition, in doses of 0.50, 0.75, 1.00, 1.50 grammes, has no sensible action on the condition of the fœtus.

4. Quinia does not seem o possess any virtue to augment the intensity of the uterine contractions.

5. The contractions, whether regular or irregular, suffer no change in their modality from any action whatever of quinia.—*New Remedies.*

ACTION OF BUTYLCHLORAL. Prof. Liebreich has made a series of experiments with butylchloral upon rabbits and the human subject, which are reported in the *Centralbl. f. d. Med. Wissensch.* In the case of human beings the following facts were noted :

To a child aged 4½ years, after trials had been made with smaller doses, 2.5 grm. of butychloral were given in sweetened water. It soon fell into a sleep, from which it could be aroused by pinching its arms, falling again into slumber as soon as the irritation ceased. Irritation of the cornea, however, had no effect, and it appeared to be entirely without sensation. This want of sensibility was noticed even when the child was roused from sleep; but the nasal mucous membrane, on the other hand, was sensitive. To lunatics 5 grm. were given, and sleep, with anæsthesia, produced while the patients remained seated upon their chairs, to such an extent were the sensibility and reflex irritability of the body maintained. Contrary to expectation, this remedy has afforded but slight relief in cases of tic-douloureux.—*Ib.*

CHRONIC INTERMITTENTS.—Dr. C. Wellers, of Columbus, Texas, writes to the *New Orleans Medical Journal:*

"The persistence with which chronic intermittents sometimes resist our best directed efforts for their cure, renders it the duty of every practitioner to make known whatever plan of treatment he may have found successful. I submit the following, which has with me proven quite successful when strictly observed by the patient.

I give a mercurial cathartic when the condition of the secretions demands it, and then quinine in full doses to interrupt the next paroxysm. I then order the following:

R.—Sulphate quinine,
Iron by hydrogen, aa gr. xc.,
Powdered myrrh,
Piperine, : - aa gr. xlv.
M. Make a mass to be divided into ninety pills.
S. One pill three times a day until *all* are taken.

In the majority of cases this would effect a cure; in some, however, it will be necessary to have the prescription refilled and continued for another month."

INFLUENCE OF ARTIFICIAL SUPPRESSION OF THE CUTANEOUS SECRETION.—Sokoloff (*Virchow's Archiv.*) experimented upon some forty-six dogs and rabbits. These animals were painted with oil and other substances, the results being as follows: The temperature usually fell. The urine showed gray and hyaline cylinders, kidney-epithelium, and young cells by which its specific gravity was increased; albumen also appeared. In one case, dropsy occurred. Diarrhœa, loss of power in the heart, cramps, sopor, and, when the coating was extensive or complete, death, in periods varying from a few hours to several days. Post-mortem examination, congestion of the brain and membranes, as well as of most of the internal organs. —*The Drug. Cir. and Chem. Gaz.*

SCROFULOUS ULCERS—RED LEAD AND CINNABAR PLASTER.—In his wards at the hospital of Saint Louis, M. Vidal has for several years made use of a plaster which he considers very efficacious in cleansing the great number of ulcers and scrofulous sores. Its composition is as follows:

| | |
|---|---|
| Diachylon plaster.......... | 26 parts. |
| Red lead...................... | 2.50 " |
| Cinnabar...................... | 1.50 " |

These ingredients are thoroughly mixed and spread upon a piece of calico like an ordinary diachylon plaster; small pieces of the plaster are used, a little larger than is sufficient to cover the ulcer. It is a very appropriate mode of treatment, and may be easily employed for a long time. M. Vidal recommends it strongly.—*London Med. Record.*

INEBRIETY.—Dr. T. D. Crothers, physcian to the New York Inebriate Asylum, in an interesting paper in the *Medical and Surgical Reporter* on the relations of melancholia to inebriety, thus concludes:

A. is a periodical inebriate from inheritance; after an attack, will disappear and wander round the country, purposeless and aimless, then recover, and return to business and friends.

B. is a periodical inebriate from obscure brain lesions. After he drinks to exhaustion and recovers in part, he is seized with a mania to look over his business with great care; nothing but a long inventory and personal inspection will satisfy him. This occurs after every paroxysm.

C. is a periodical inebriate from hardship and mental trouble. After the paroxysm is over, he walks the floor and is dangerously sensitive; will not bear confinement or contradiction, for a long time.

These symptoms are frequent in chronic cases, and are similar to melancholia. We conclude that melancholia may not be noticed before inebriety is developed, but it appears in many forms after inebriety comes on.

The third statement, viz., that the presence and prominence of melancholia with inebriety indicates profound disorder and complications, with a special prognosis and treatment, is evident beyond question.

The following seems to be the general course and termination in all these cases:

1. All the symptoms increase into chronicity, ending in dementia or chronic alcoholism and death.

2. They may merge into acute disease, which will be localized in some organ, terminating in recovery or death.

3. The presence of both melancholia and inebriety may continue without marked change for a long time, then suddenly one or the other becomes intensified into great activity, or both disappear.

The first course or termination is very common. In the second, acute attacks of gastritis, hepatitis, various diseases of the kidneys, and often pneumonia, follow, and if they do not end fatally, the melancholia and inebriety disappear in many cases.

In the third class the cases are not closely observed, but, without doubt, they are numerous: a class of patients who drink to intoxication at long intervals, also suffering from melancholia and depression; this goes on without change for years, then they become demented or helpless drunkards, or from obscure cause they recover.

These cases are seldom recognized until the last stages, but, nevertheless, they possess the same symptoms, only in a less degree, as the most chronic cases.

To term this early stage a vice, because of its obscurity, is a strange misnomer, arising from profound ignorance.

The prognosis and treatment depends upon the history of each case and its various complications. In general terms, the marked presence of melancholia with inebriety is significant of general degeneration. When inebriety seems to follow melancholia the prognosis is also bad, and the treatment by prolonged residence and abstinence from the exciting causes, in an asylum, under medical care, gives the only promise of help.

If these statements are correct, the early stages of these two affections are the same, and when the associations continues the case is serious. The conclusions may be summarized as follows :—

*First.* Inebriety and melancholia are identical in their early manifestations.

*Second.* They may follow each other, as both cause and effect.

*Third.* Their course and termination are alike, and they are subject to similar variations and complications.

*Fourth.* Both of these disorders are stages or phases of insanity with similar prognosis, and requiring the same general treatment.

Chloral in Eclampsia.—Dr. J. Masten, of Whitfield, Pa, reports to *Med. and Surg. Reporter*, a case of Eclampsia successfully treated with chloral hypodermically. He remarks:

I found my patient with strong convulsive movements of the facial muscles and muscles of the extremities, and was informed by her attendants that this was the second, and a very severe convulsion. Failing to procure either chloroform or ether, I felt that I was in very close quarters, with a responsible and dangerous case on my hands, and that the pulse, which was very weak and fluttering, forbade the use of the lancet.

Being thrown entirely upon my own resources, and considering myself justified in using any remedy that offered a hope of success, I immediately dissolved what I judged to be ten grains of chloral hydrate in a small quantity of water, and injected it subcutaneously in the left leg. The convulsive movements soon ceased, and I was gratified beyond expression to see my patient begin to rally, although nearly half an hour passed before she could control the muscles of deglutition, after which I administered the chloral, in combination with bromide of potash, about ten grains each, every hour. There was complete blindness for over an hour, but no symptoms of a return of the convulsions until 7 A.M., of the 24th. She then informed me that the pain in her stomach was returning, and that she was getting blind again, precursor of a third convulsion. I commenced crowding the chloral and bromide, and the attack was warded off.

There was no return of the convulsions, nor any symptoms, after this period. It was not until three days after confinement that she realized that her labor had taken plaee. I saw the patient on the second day of June, and found her getting along fully as well as in previous confinements.

I attribute the good results in this case to the prompt admistration of the chloral hypodemically. I was unable to examine the urine for albumen, from the fact that, from the first convulsion, until forty-eight hours after, there was complete suppression of urine.

Something New About Oxygen.—Recent investigations have disclosed the singular fact that oxygen under high pressure rapidly destroys all living beings and organic compounds. All the varied phenomena of fermentation, in which the chemical action depends upon the presence of living organisms, are completely arrested by the action of compressed oxygen, even if exerted for only a brief time; while fermentations due to dissolved matter, like diastase, perfectly resist its influence. M. Bert, to whom this curious discovery is due, has found a practical application of it in the field of physiological research. The ripening of fruits is arrested by exposure to compressed oxygen, and hence it must arise from cellular evolution. The poison of the scorpion, on the other hand, whether liquid, or dried and redissolved in water, entirely resists the action of the compressed gas. Such poisons evidently owe their power to chemical compounds akin to the vegetable alkaloids. Fresh vaccine matter, subjected for more than a week to oxygen under a pressure equal to fifty atmospheres, retained its virtue; from which it would appear that the active principle in vaccine matter is not certain living organisms or cells, as some have supposed. The virus of glanders, after similar treatment, quickly infected horses inoculated with it; and carbun–

cular blood, though freed from bacteria, was found to retain its dangerous properties after the same test. These must, therefore, be put in the same class with vaccine matter.

If these results are confirmed by further investigations, the discovery is certainly a most important one, and will lead to the settlement of many disputed questions in physiological chemistry.—*Jour. of Ghem.*

CHOICE OF SEDATIVES FOR THE VERY YOUNG.—Dr. Stokoe (Guy's Hospital Reports for 1876) says: "If we purpose giving a sedative to the very old or very young, we must be cautious, especially in using any of the preparations of opium, often cumulative in their effects. As a consequence of this, for some years past I have trusted almost entirely to sedatives other than opiates in treating child ren in their first septennate, and I have seen no reason to believe that any want of success has ensued from this exclusiveness. That such a precautionary measure is not altogether uncalled for, has been impressed upon me by my experience of the method of medication adopted by the more ignorant (including nurses and nursery-maids), whose frequent habit is to increase the prescribed dose sevenfold, or to repeat it with undue persistence, if it should fall short of the expected effect; with what result may be conceived when two or three minims of laudanum have been ordered for an infant. With potassic bromide and conium for the various morbid conditions incidental to teething; chloroform for administration during the paroxysm of a conclusive attack; chloral for those derangements in which insomnia is the prevailing symptom; aconite for inflammations, fevers, and feverishness generally; belladonna and hyoscyamus for many visceral disorders of a painful or obstinate nature, and combinations of these and other drugs to soothe coughs and the innumerable aches and pains of neuralgic, myalgic, or rheumatic origin—to say nothing of a host of external sedative applications, many of which are very potent—we need be under no apprehension lest we should be incapable of coping with the assaults of disease in children as effectually as we could do with one more weapon in our repertory."—*Drug Cir.*

